# Evaluation of HIV-specific T-cell responses in HIV-infected older patients with controlled viremia on long-term antiretroviral therapy

**DOI:** 10.1371/journal.pone.0236320

**Published:** 2020-09-17

**Authors:** Nicole E. Behrens, Anne Wertheimer, Maria B. Love, Stephen A. Klotz, Nafees Ahmad

**Affiliations:** 1 Department of Immunobiology, The University of Arizona Health Sciences Center, Tucson, AZ, United States of America; 2 Department of Medicine, The University of Arizona Health Sciences Center, Tucson, AZ, United States of America; 3 College of Medicine, and Bio5 Institute, The University of Arizona Health Sciences Center, Tucson, AZ, United States of America; Duke University, UNITED STATES

## Abstract

HIV-infected older individuals may have a diminished immune response because of exhaustion/immune aging of T-cells. Therefore, we have investigated HIV-specific CD4 and CD8 T-cell responses in 100 HIV-infected patients (HIV^+^) who have aged on long-term antiretroviral therapy (ART) and achieved controlled viremia (mostly undetectable viral load; 92 patients with <20 to <40 HIV RNA copies/mL and 8 <60 to <100) and improved CD4 T-cell counts. We show that the median frequencies of HIV-specific CD4^+^ and CD8^+^ IFN-γ T-cells were higher in HIV^+^ than uninfected individuals (HIV^-^), including increasing levels of IFN-γproduced by CD4^+^ T-cells and decreasing levels by CD8^+^ T-cells with increasing CD4 T-cell counts in HIV^+^. No correlation was found between T-cell responses and varying levels of undetectable viremia. HIV-specific TNF-α made by CD8^+^ T-cells was higher in HIV^+^ than HIV^-^, including decreasing levels with increasing CD4 T-cell counts in HIV^+^. Furthermore, the CD8^+^ T-cell mediators, CD107a and Granzyme-B, were higher in HIV^+^ than HIV^-^, and decreased with increasing CD4 T-cell counts in HIV^+^. Remarkably, HIV-specific CD8 T-cells produced decreasing levels of IFN-γwith increasing age of HIV^+^, including decreased levels of CD107a and Granzyme-B in older HIV^+^. However, HIV-specific CD8^+^ T-cells produced increasing levels of TNF-α with increasing age of the HIV^+^, suggesting continued inflammation. In conclusion, HIV^+^ with controlled viremia on long-term ART and with higher CD4 T-cell counts showed reduced HIV-specific CD8 T-cell responses as compared to those with lower CD4 T-cell counts, and older HIV^+^ exhibited decreasing levels of CD8 T-cell responses with increasing age.

## Introduction

Despite the devastating effects of human immunodeficiency virus type 1 (HIV) infection on host immunity, infected adults mount a robust immune response against HIV antigens [1]. However, HIV-infected older individuals may have a less protective immune response because of reduced immunity as a result of immune aging or immunosenescence [2]. Immunosenescence contributes to increased susceptibility of older people to new infections, frequent recurrences to chronic and latent infections, and poor response to vaccination mainly because of declined T-cell functions [2]. Several studies have shown that older individuals have a diminished CD4^+^ T-cell function [3] and produce less IL-2 [4], which reduces the interaction between CD4^+^ and CD8^+^ T-cells resulting in less effective cytotoxic T-lymphocytes response [5]. In the era of expansion of antiretroviral drugs, many HIV-infected patients (HIV^+^) are successfully treated with long-term antiretroviral therapy (ART) resulting in controlled viremia (undetectable to very low viral load), improved CD4 T-cell counts, reduced incidence of opportunistic infections and increased life expectancy [6,7].

Some HIV-infected patients with controlled viremia and unrestored CD4 T-cell counts experience increased T-cell activation, inflammation and coagulation known as residual immune dysregulation syndrome (RIDS) associated with increased morbidity and mortality [8,9]. Immunocompromised patients, HIV immune non-responders, RIDS patients and older individuals have increased levels of circulating inflammatory cytokines, immune activation of CD8^+^ T-cells, and immunosenescence [8,9] probably because of low level persistent viral replication [10–12]. In HIV^+^ patients on long-term ART, HIV persists in resting memory CD4^+^ T-cells as stable latent reservoirs, which play a major role in chronic activation of HIV-specific cytotoxic CD8^+^ T-cells [13–15]. Nevertheless, HIV-specific T-cell responses in these HIV^+^ older patients with controlled viremia and improved CD4 T-cell counts are not fully explored.

Cytotoxic T-lymphocytes (CTL) react with *env*, *pol*, *gag* and regulatory and accessory gene products [5]. Envelope specific CTL reactivity is seen in nearly all infected individuals, but decreases with disease progression [16]. In addition, Gag-specific CTL are associated with lower viral loads and decreased risk of HIV disease progression [17–19]. Several studies have analyzed HIV-specific CTL in HIV-infected patients on ART and showed a significant decline in CTL over time [20,21]; however these CTL do not fully disappear and can be detected many years after effective ART [22–24]. More importantly, the protective roles of CTL in HIV^+^ patients who have aged on long-term ART and experienced immunosenescence have not been elucidated. Understanding HIV-specific T-cell responses in these aging and older patients may provide information for developing preventive and treatment strategies leading to a possible cure. We, therefore, sought to determine HIV-specific CD4^+^ and CD8^+^ T-cell responses in HIV^+^ older patients with controlled viremia and increasing CD4^+^ T-cell counts due to long-term ART and their increasing age.

Here we show that the CD4^+^ and CD8^+^ T-cells from HIV^+^ older patients with controlled viremia (92% of the patients had <20 to <40 HIV RNA copies/mL and 8% <60 to <100) due to long-term ART produced proinflammatory cytokines, such as IFN-γ and TNF-α as well as markers of cytotoxic CD8 T-cell mediators, CD107a and Granzyme-B, which were modulated with increasing CD4 T-cell counts and age of HIV^+^ patients.

## Materials and methods

### Human subjects and cell preparation

This study was approved by the Institutional Review Board of the University of Arizona, Tucson, Arizona. Informed and signed consent forms were obtained from all participants in the study. All methods were performed in accordance with relevant guidelines and regulations approved by the University of Arizona Biosafety Committee. The HIV-infected patients’ cohort (HIV^+^) consisted of 100 clinically diagnosed HIV-infected aging patients with a median age of 53 years. However, we included HIV-infected patients’ age ranged from 21 to 81 years for age comparison. The HIV-infected patients cohort had a median CD4 T-cell counts of 528 cells/mL and CD8 T-cell counts of 645 cells/mL belonging to different ethnicities such as White, Hispanic, African American, Asian, American Indian, and Mexican American (84 males and 16 females) receiving health care and antiretroviral therapy (ART) for at least 3 years and up to 30 years at the Petersen HIV Clinic, Division of Infectious Disease, Banner University Medical Center, Tucson, Arizona. The HIV^+^ patients mostly had undetectable to some with very low viral load, including 81 patients with viral load of <20 HIV RNA copies/mL, 11 patients <40 copies/mL, 3 patients <60 copies/mL, 3 patients <80 copies/mL and 2 patients <100 copies/mL. While several HIV clinical labs consider <50 HIV RNA copies/mL as undetectable viral load, DHHS/CDC considers <200 HIV copies/mL as undetectable viral load. The HIV-1 in our HIV-infected patients belonged to Subtype B. The HIV-uninfected control cohort (HIV^-^) included 75 healthy age-matched (median age: 53 years, CD4 T-cell counts: 958 cells/mL, and CD8 T-cell counts: 338 cells/mL) self-reported HIV-uninfected controls. The number of participants per age group were as follows: 21–30 years: HIV^+^ = 4, HIV^-^ = 8; 31–40 years: HIV^+^ = 8, HIV^-^ = 7; 41–50 years: HIV^+^ = 29, HIV^-^ = 20; 51–60 years: HIV^+^ = 37, HIV^-^ = 21; 61–70 years: HIV^+^ = 18, HIV^-^ = 15; and 71–81 years: HIV^+^ = 4, HIV^-^ = 4. The demographics and laboratory parameters of HIV-infected and uninfected cohorts are summarized in Table 1. Blood samples were drawn into 4 10 mL sodium heparin or 4 10 mL CPT mononuclear cell preparation sodium heparin tubes BD Vacutainer tubes (BD, Sunnyvale, CA). Blood samples drawn from a single time point mostly the first draw of the individuals enrolled in our study were processed at the University of Arizona Biorepository Laboratory per their protocols and peripheral blood mononuclear cells (PBMC) were cryopreserved for future analysis. One K2 EDTA tube was collected to determine complete blood counts, using an A^c^-T 5diff CP machine (Beckman Coulter, Pasadena, CA).

**Table 1:** HIV-infected (HIV^+^) and uninfected (HIV^-^) cohorts demographics, clinical and laboratory parameters.

Cohort	Number of subjects (n)	Age (yrs)	Median age (yrs)	Gender (n)	CD4 count (cells/mL)	Median CD4 count (cells/mL)	CD8 count (cells/mL)	Median CD8 count (cells/mL)	Viral load HIV RNA (copies/mL	Ethnicity
HIV^+^	100	22–81	53	F:16 M:84	70–1592	528	149–1680	645	<20 - <100	A, AA, MA, O, H, W
HIV^-^	75	23–7 5	53	F:35 M:40	472–1845	958	114–786	338	N/A	AA, AI, H, W

F: Female, M: Male, A: Asian, AA: African American, AI: American Indian, MA: Mexican American, O: Other, H: Hispanic, W: White, N/A: Not applicable

### Flow cytometry (FCM)

The cryopreserved PBMC (1-3x10^6^/well) were stained with LIVE/DEAD Fixable Dead cell Stain- AQUA (Invitrogen), T-cell markers and cytokines markers in various combinations as described in our previous study [25]. Briefly for this study we used: CD3^+^–BV570 (BioLegend), CD4^+^–APC (eBioscience), CD8β^+^–ECD (Beckman Coulter), CD107a– PE-Cy7 (BioLegend), IFN-γ–APCe780 (eBioscience), Granzyme-B–A700 (BD Pharmogen), TNF-α–PE (eBioscience).

Cryopreserved PBMC were thawed and 1x10^6^ viable cells/well were plated for unstimulated and PMA-stimulated wells, and 3x10^6^ cells/well for the peptide pool stimulated wells in 5% X-VIVO 15 media (X-VIVO 15 + 5% male human AB serum) and rested for 16–20 hours in a 37°C incubator with 5% CO_2_. Post thaw viability was determined using nuclear staining dye (AO/PI) (Nexcelom Bioscience LLC., Lawrence, MA) and read on a Nexcelom Bioscience cellometer. Following overnight resting, peptide wells were stimulated with 200ng/well each of Subtype B HIV Gag (HIV-1 potential T-cell epitopes (PTE) Gag Peptide Pool) and HIV Env (HIV-1 PTE Env Peptide Pool) peptide pools [26,27] (NIH AIDS Reagent Program) for 4 hours at 37°C and 5% CO_2_. These peptide (15 amino acids in length) pools were chosen for their intended use in T-cell assays [26,27]. Their primary listed use is for intracellular cytokine staining by flow cytometry allowing for measurements of HIV-1 specific responses [27]. PMA stimulated wells were challenged with a 1:500 dilution of PMA-Ionomycin (eBioscience) for 2 hours at 37°C and 5% CO_2_. Prior to running final reactions, time trials were conducted for determining the optimal stimulation time for PMA and Peptide stimulations and ensuring that the reactions were non-toxic and did not change extracellular marker expression. All CD8^+^ stained wells were also stained with CD107a for 2 hours during the stimulation incubations. All wells were incubated for 2 hours with a combination of 1X Monensin Solution (2μM/ml) and 1X Brefeldin A Solution (3.0μg/ml) (eBioscience). The antibody staining protocol is described in our previous study [25]. Samples were analysis on the BD LSR II instrument, using DiVa acquisition (BDIS, Mountain View, CA). All data was collected using set optimized standard voltages and calibration was performed daily using Rainbow beads (BD Bioscience).

**Fig 1:**
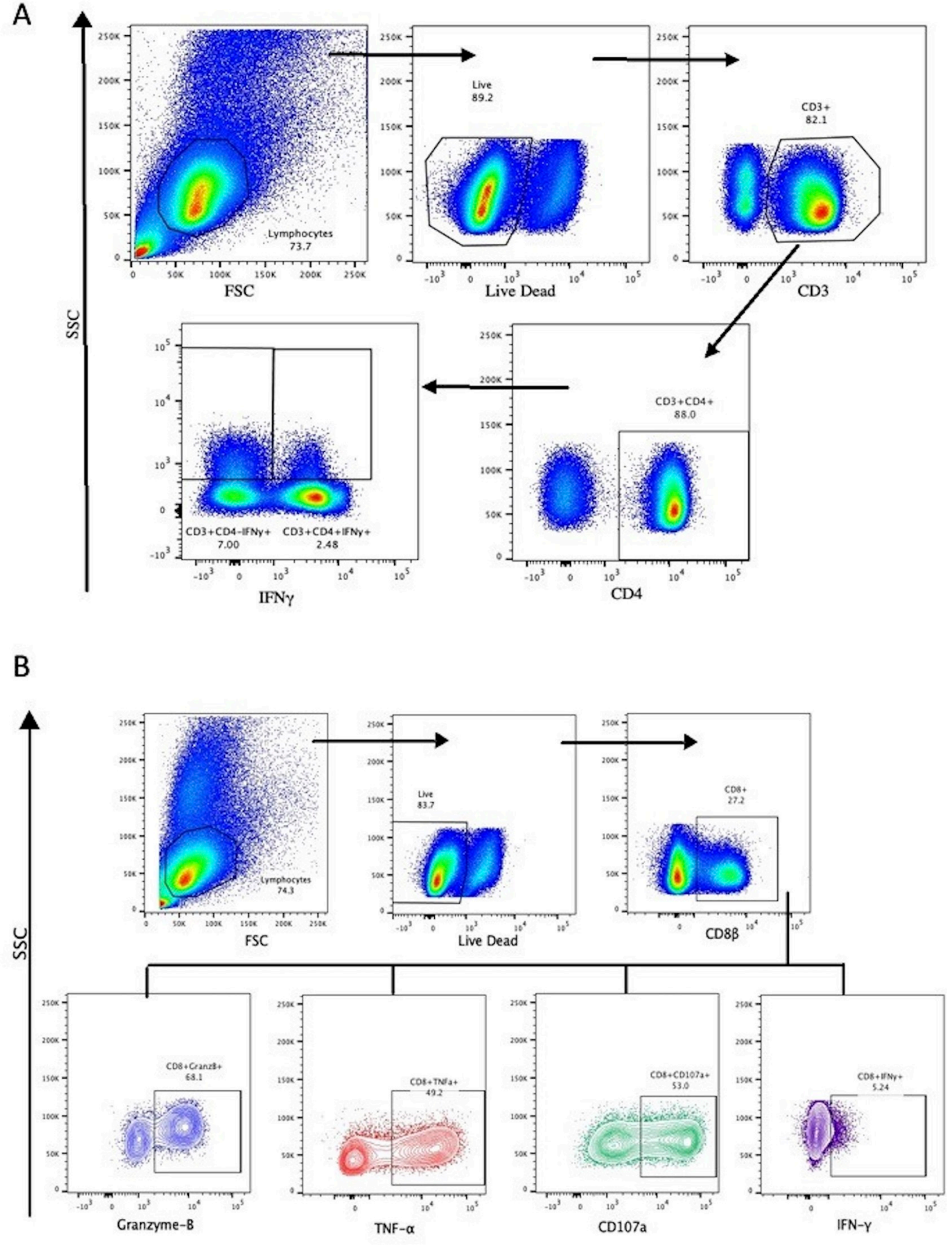
CD4^+^ and CD8^+^ flow cytometry gating strategy. (A) CD4^+^ gating strategy beginning with isolation of lymphocyte subset followed by Live/Dead staining. Once in the Live subset, gating for CD3^+^ and CD4^+^ and followed by intracellular cytokine staining for IFN-γ. (B) CD8^+^ gating strategy beginning with isolation of lymphocyte subset followed by Live/Dead staining. Live cells were then gated for CD8β^+^ T-cells, and then gating of intracellular Granzyme-B, TNF-α, IFN-γ, and membrane protein, CD107a.

All samples were analyzed using FlowJo version 10 analysis software (TreeStar Inc., Ashland, OR). Samples were first gated for total lymphocyte population followed by lymphocyte viability. For CD4^+^ T-cell populations, the viable lymphocyte population was then gated through CD3^+^ followed by CD4^+^, once within the CD3^+^CD4^+^ populations samples were gated for CD4^-^IFN-γ or CD4^+^IFN-γ. For CD8^+^ T-cell populations, the viable lymphocyte population was gated through CD8β followed by IFN-γ, TNF-α, CD107a, or Granzyme-B. The positive and negative gates for cytokine production were determined using preset standard positive and negative gates, created utilizing an over lay of single stained cells and stained beads to obtain true positive and negative gates without influence from other fluorochromes. These positive and negative gates were confirmed by utilizing an all stain stimulated control that was overlaid onto the single stained control cells to ensure that the compensation or multiple fluorochromes did not influence the position of the positive and negative gates. The gating strategy is shown in Fig 1. The process of culturing and stimulating cells in any live cell experiment results in some cells loss and proliferation that cannot be accurately estimated, thus appropriate controls are utilized for normalization throughout the process. The cell frequencies obtained from Flow Jo version 10 software, which are estimates of cell populations.

### Statistical analysis

All cell population totals were graphed using median values with 95% confidence intervals. An unpaired, non-parametric Mann-Whitney test was used to determine statistical significance between different populations, including treatment groups HIV^+^ vs HIV^-^, or between different stimulation conditions. The statistical values for the non-parametric Mann-Whitney test were reported as *p<0.05, **p<0.01, ***p<0.001, ****p<0.0001. A linear regression model was used for all linear plots comparing a population to CD4 T-cell counts and age of the subjects. All statistical significance was obtained by comparing the slope of the population to a slope of zero. In the event both linear regression slopes were significantly non-zero, the slopes were compared to each other to determine if they were significantly different. Software used was Graphpad Prism version 7.

## Results

### HIV-specific IFN-γ and TNF-α production by CD4^+^ and CD8^+^ T-cell in HIV^+^ aging patients with controlled viremia on long-term ART

**Fig 2:**
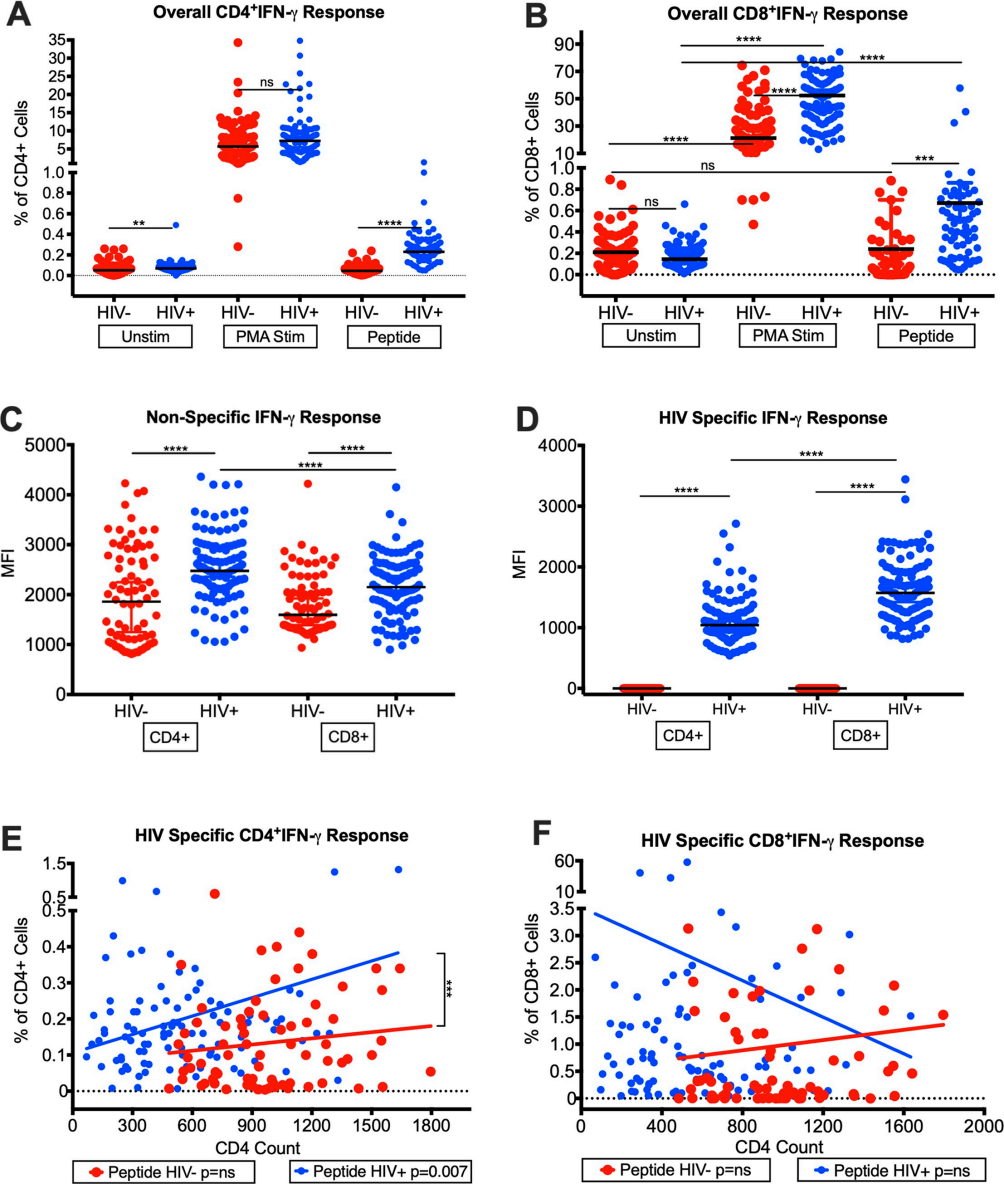
Intracellular production of IFN-γ by CD4^+^ and CD8^+^ T-cells from HIV-infected patients with controlled viremia on ART. Frequencies of intracellular CD4^+^ IFN-γ T-cells (A) and CD8^+^ IFN-γ T-cells (B) under three treatment conditions; unstimulated, PMA stimulated and HIV-Gag/Env peptide pool stimulated in HIV^+^ and HIV^-^ cohorts. Median fluorescent intensity (MFI) of PMA stimulated (C) and HIV Gag/Env peptide pool stimulated (D) intracellular IFN-γ production by CD4^+^ and CD8^+^ T-cells from HIV^+^ and HIV^-^ cohorts. Frequencies of HIV-Gag/Env peptide pool simulated intracellular IFN-γ in CD4^+^ (E) and CD8^+^ (F) T-cell populations with increasing CD4 T-cell counts in HIV^+^ and HIV^-^ cohorts (n = 100 HIV^+^, n = 75 HIV^-^) (*p<0.05, **p<0.01, ***p<0.001, ****p<0.0001).

To determine HIV-specific CD4^+^ and CD8^+^ T-cell responses in HIV^+^ with controlled viremia (mostly undetectable viral load of <20 to <40 HIV-1 RNA copies/mL and some with very low of <60 to <100) and mostly improved CD4 T-cell counts (median CD4 T-cell counts: 528 cells/mL), we measured IFN-γ and TNF-α production in 100 HIV^+^ aging patients (median age: 53 years) and compared with 75 HIV-uninfected controls (HIV^-^) (median age: 53 years and median CD4 T-cell counts: 958 cells/mL). The cell frequencies are represented as median values. First, we determined the background and PMA stimulated levels of IFN-γ and TNF-α produced by CD4 and CD8 T cells to show that these cells are functional by making these cytokines. We found that the background levels of median frequencies of CD4^+^ T-cells expressing IFN-γ were higher in HIV^+^ (0.069%) than HIV^-^ (0.051%) p = 0.0021 (Fig 2A), whereas the median frequencies of CD8^+^IFN-γ T-cells were lower in HIV^+^ (0.145%) compared with HIV^-^ (0.21%) (Fig 2B). Upon stimulation with PMA-Ionomycin, both HIV^+^ and HIV^-^ showed an increase (p<0.0001) in their frequencies of CD4^+^IFN-γ and CD8^+^IFN-γ T-cells as compared to the background levels (Fig 2A and 2B). However, the median frequencies of CD4^+^IFN-γ T-cells were higher in HIV^+^ (7.215%) compared with HIV^-^ (5.69%), while frequencies of the CD8^+^IFN-γ T-cells were significantly higher (p<0.0001) in HIV^+^ (52.35%) compared with HIV^-^ (21.2%) (Fig 2A and 2B). Additionally, we determined the median fluorescence intensity (MFI) and found that MFI for PMA stimulated CD4^+^IFN-γ (MFI: 2477) and CD8^+^IFN-γ (MFI: 2148) T-cells were higher (p<0.0001) in HIV^+^ compared with HIV^-^ (CD4^+^ MFI: 1859, CD8^+^ MFI: 1597) (Fig 2C). Interestingly, the MFI of CD4^+^IFN-γ T-cells were significantly higher (p<0.0001) than CD8^+^IFN-γ T-cells in HIV^+^ (Fig 2C) even though the median CD4 T-cell population was lower compared with CD8 T-cell counts (median CD4 T-cell counts: 528 cells/mL, median CD8 T-cell counts of 645 cells/mL).

We then examined the frequencies of HIV-specific CD4^+^IFN-γ and CD8^+^IFN-γ T-cells in response to the combined HIV Gag and Env peptide pools [26,27]. We found that the combined Gag/Env peptide pool stimulated T cells from HIV^+^ showed a significant increase in the frequencies of CD4^+^IFN-γ (0.23%, p<0.0001) (Fig 2A) and CD8^+^IFN-γ (0.67%, p<0.001) (Fig 2B) T-cells over unstimulated cells from HIV^+^ (CD4^+^: 0.045%; CD8^+^: 0.17%). As expected, there was no difference in unstimulated or Gag/Env peptides stimulated CD4^+^ (Fig 2A) and CD8^+^ T-cells (Fig 2B) for IFN-γ production from HIV^-^. These data demonstrate that CD4^+^ and CD8^+^ T-cells from our HIV^+^ with controlled viremia still produce IFN-γ upon stimulation with HIV-specific antigens. Moreover, the MFI for HIV Gag/Env peptide specific CD8^+^IFN-γ T-cells (1573 MFI) for HIV^+^ was significantly higher (p<0.0001) than CD4^+^IFN-γ T-cells (1042 MFI) (Fig 2D), suggesting that CD8^+^ T-cells produced more IFN-γ to control the presence of residual HIV in patients with controlled viremia.

Next, we determined the frequencies of CD4^+^ and CD8^+^ T-cells producing IFN-γ in response to HIV Gag/Env peptides with increasing CD4^+^ T-cell counts of HIV^+^. We found a significant increase in the frequencies of CD4^+^IFN-γ T-cells in HIV^+^ with increasing CD4 T-cell counts (Fig 2E), with a significant difference (p<0.0001) in the rise of the slope between the peptide stimulated HIV^+^ compared with both unstimulated HIV^+^ and peptide stimulated HIV^-^, with the HIV^+^ peptide response increasing with a faster slope with increasing CD4 T-cell counts (Fig 2E). Interestingly, HIV^+^ patients with lower CD4 T-cell counts also had fewer proportion of CD4 T cells producing IFN-γ. On the other hand, the frequencies of CD8^+^IFN-γ T-cells from HIV^+^ in response to Gag/Env peptide stimulation decreased with increasing number of CD4 T cell counts in our virologically controlled HIV^+^ patients with undetectable to very low viral load (Fig 2F), demonstrating that the reduced levels of HIV-specific CD8 T cells correlates with increasing CD4 T cell count in HIV^+^. While Fig 2B (middle bars) shows that the median frequencies of PMA stimulated CD8^+^IFN-γ^+^ of HIV^+^ were significantly higher in HIV^+^ than HIV^-^, these non-specific IFN-γ^+^ production were unchanged and did not show decline with increasing CD4 T cell counts (S1C Fig), because of significant non-specific stimulation by PMA of about 50% of CD8 T cells in HIV^+^ patients.

We also determined the frequencies of CD4^+^ and CD8^+^ T-cells producing IFN-γ in response to HIV Gag/Env peptides with increasing HIV RNA copies/mL of the undetectable to very low viral load in our 100 HIV^+^ patients (81 patents had <20, 11 <40, 3 <60, 3 <80 and 2 <100 HIV RNA copies/mL). We found no correlation between HIV-specific CD4 and CD8 T cells producing IFN-γ and the varying level of HIV RNA copies of the undetectable to very low viral load in our HIV^+^ patients as shown in S1A and S1B Fig. All our HIV^+^ patients had undetectable to very low viral load due to on long-term ART, which is most likely below the threshold to cause any further stimulation of T cells. The correlation between viral load and CD4 T cell counts of our 100 HIV+ patients is shown in S1D Fig., which shows different levels of restoration of CD4 T cells counts.

**Fig 3:**
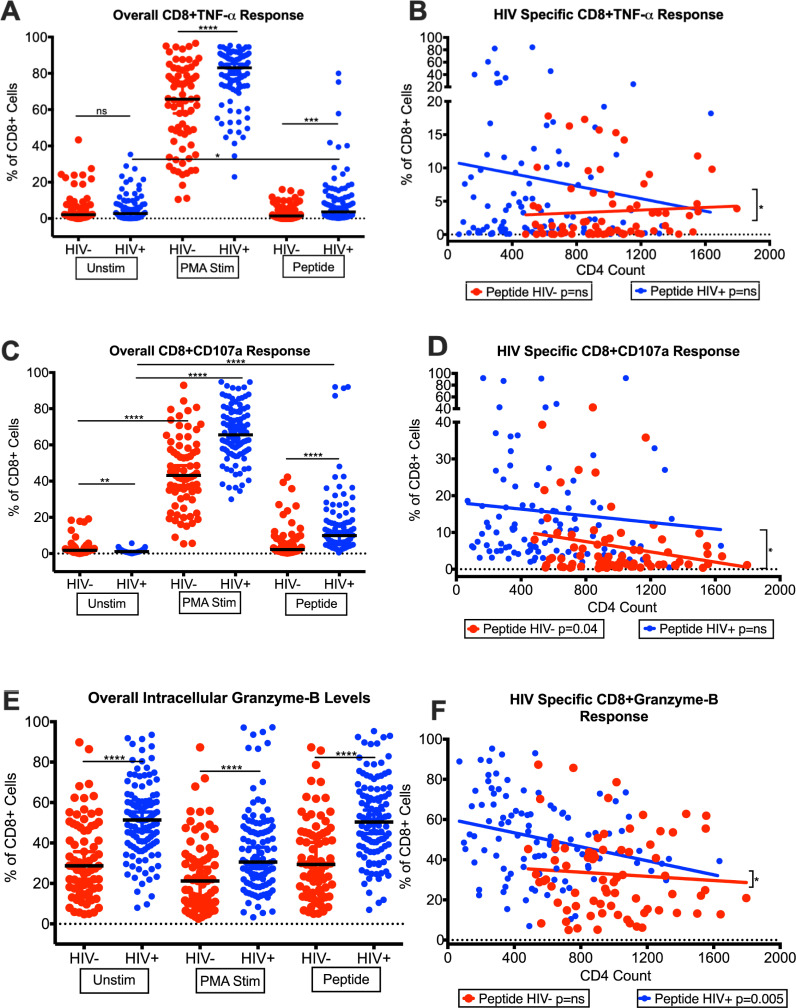
Intracellular production of TNF-α, CD107a and Granzyme-B by CD8^+^ T-cells from HIV-infected patients with controlled viremia on ART. Frequencies of intracellular TNF-α (A) and CD107a (C) and Granzyme-B (E) production by CD8^+^ T-cells under three treatment conditions; unstimulated, PMA stimulated and Gag/Env peptide pool stimulated in HIV^+^ and HIV^-^ cohorts. Frequencies HIV Gag/Env peptide pool simulated and unstimulated intracellular TNF-α (B), CD107a (D) and Granzyme-B (F) production by CD8^+^ T-cell with increasing CD4 T-cell counts in HIV^+^ and HIV^-^ cohorts. (n = 100 HIV^+^, n = 75 HIV^-^) (*p<0.05, **p<0.01, ***p<0.001, ****p<0.0001).

Finally, we evaluated the frequencies of HIV-specific and non-specific TNF-α producing CD8^+^ T-cells. The background levels of median frequencies of CD8^+^TNF-α T-cells were slightly higher in HIV^+^ (2.7%) compared with HIV^-^ (2.07%) (Fig 3A). Upon stimulation with PMA, both HIV^+^ (83%) and HIV^-^ (65.75%) showed a significant increase (p<0.0001) in CD8^+^TNF-α T-cell frequencies compared with unstimulated levels, including a higher frequencies (p<0.0001) of CD8^+^TNF-α T-cells in HIV^+^ than HIV^-^. More importantly, the HIV Gag/Env peptides challenged CD8^+^ T-cells responded in a significant increase in the frequencies of TNF-α producing cells in HIV^+^ (3.62%) compared with HIV^-^ (1.4%) (p<0.0001) and the unstimulated cells in HIV^+^ (2.7%) (p = 0.0151) (Fig 3A). Furthermore, HIV Gag/Env peptide specific response in HIV^+^ showed a decreasing frequency of CD8^+^TNF-α T-cells with increasing CD4 T-cell counts in HIV^+^ (Fig 3B), suggesting decrease inflammation because of improved CD4 T-cell counts. It is important to note that the levels of HIV-specific CD8 TNF-α were higher in HIV^+^ with lower CD4 T cell counts (Fig 3B).

### Evaluation of CD8^+^ T-cells degranulation and lytic functions by measuring CD107a and Granzyme-B production in HIV^+^ aging patients with controlled viremia on long-term ART

We determined the expression of membrane protein CD107a on CD8^+^ T-cells, which has been reported as a marker for degranulation on lymphocytes, including CD8^+^ T-cells and for its effects on lysosomal granule exocytosis [28]. The cell frequencies are shown as median values. In our analysis, the background frequencies of CD8^+^CD107a T-cells were lower in HIV^+^ (1.055%) than HIV^-^ (1.76%) (Fig 3C). Upon stimulation with PMA, we saw a significant increase (p<0.0001) in the frequencies of CD8^+^CD107a T-cells in both HIV^+^ and HIV^-^, however, the cells from HIV^+^ showed a more robust response (p<0.0001) with a median frequency of 56.55% compared with 43.1% in HIV^-^ (Fig 3C). When stimulated with the combined HIV-Gag/Env peptide pool, the HIV^+^ showed a significant increase in the frequencies of CD8^+^CD107a T-cells (9.896%) compared with HIV^+^ (unstimulated,1.055%) and HIV^-^ (peptide stimulated, 2.22%) as shown in Fig 3C. More importantly, we saw a significant decrease (p = 0.045) in Gag/Env peptide stimulated CD8^+^CD107a T-cells frequencies with increasing CD4 T-cell counts in HIV^+^ compared with peptide stimulated cells from HIV^-^ (Fig 3D). In addition, there was a significant difference between the slopes of the peptide stimulated cells from HIV^+^ and unstimulated cells from HIV^+^ (p = 0.041) and peptide stimulated cells from HIV^-^ (p = 0.043) (Fig 3D). These data also suggest that CD8 T-cell cytotoxic response decreased in HIV^+^ with improved CD4 T-cells counts.

We next examined the intracellular expression of Granzyme-B in the CD8^+^ T-cells of our HIV^+^ with controlled viremia. Granzyme-B is found in the granules of cytotoxic T-cells and natural killer (NK) cells and expressed at higher levels in HIV^+^ patients with uncontrolled viremia leading to a rapid death of CD4^+^ T-cells [28,29]. Our data shows that the frequencies of CD8^+^ T-cells producing Granzyme-B were significantly elevated in our HIV^+^ (51.4%) compared with HIV^-^ (28.7%) (Fig 3E). However, the frequencies of CD8^+^Ganzyme-B T-cells significantly decreased (p = 0.047) with increasing CD4 T-cell counts in HIV^+^ compared with HIV^-^ (Fig 3F). This data supports the notion that in those HIV^+^ that have achieved improvement of CD4 T-cell counts due to controlled viremia on ART, the need for cytotoxic CD8^+^ T-cells to release Granzyme-B also decreased.

### Evaluation of HIV-specific T-cell responses with increasing age of HIV-infected patients with controlled viremia on long-term ART

**Fig 4:**
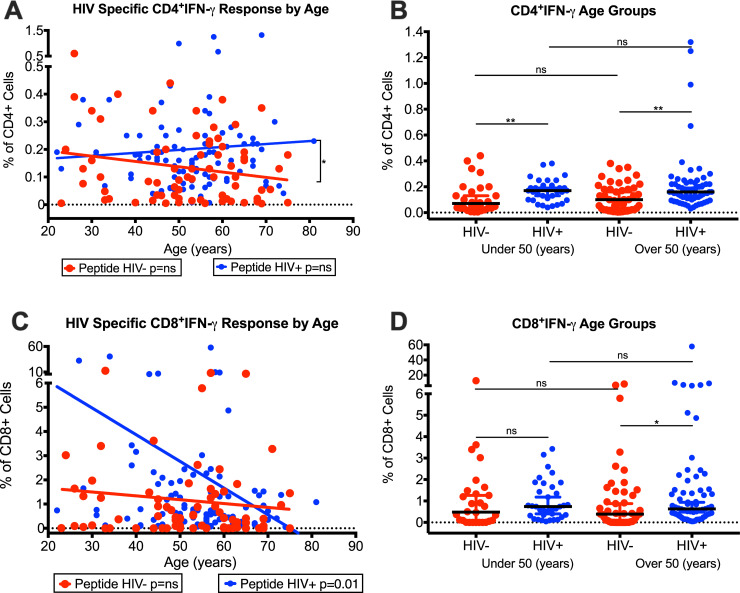
Age related changes in intracellular IFN-γ production by CD4^+^ and CD8^+^ T-cells from HIV-infected patients with controlled viremia on ART. Frequencies of HIV Gag/Env peptide pool simulated and unstimulated intracellular IFN-γ in CD4^+^ (A) and CD8^+^ (C) T-cells with increasing age of HIV^+^ and HIV^-^ cohorts. Frequencies of HIV Gag/Env peptide pool simulated intracellular IFN-γ in CD4^+^ (B) and CD8^+^ (D) T-cells in 2 age groups; under 50 years (n = 41 HIV^+^, 35 HIV^-^) and over 50 years (n = 59 HIV^+^, 40 HIV^-^), using interquartile bell curve median in HIV^+^ (n = 100) and HIV^-^ cohorts (n = 75 HIV^-^) (*p<0.05, **p<0.01, ***p<0.001, ****p<0.0001).

HIV-specific CD4 and CD8 T-cell responses in our HIV^+^ with controlled viremia and improved CD4 T-cell counts were determined with increasing age of HIV^+^. The response to HIV-Gag/Env peptide pool stimulated CD4^+^ T-cells showed an unchanged frequencies of CD4^+^IFN-γ T cells (Fig 4A), whereas CD8^+^IFN-γ T cells frequencies were significantly (p = 0.01) decreased (Fig 4C) with increasing age of the HIV^+^ patient cohort. We also compared the HIV-specific CD4^+^IFN-γ and CD8^+^IFN-γ T cells separated into two groups of under 50 years and over 50 years of age, using interquartile bell curve median in HIV^+^ and HIV^-^ cohorts as shown is Fig 4B and 4C. The HIV-specific CD4^+^IFN-γ (Fig 4B) showed a significant increase (p<0.001) the median frequencies in the HIV^+^ compared with HIV^-^ in both groups under 50 (HIV^+^: 0.17%, HIV^-^: 0.07%) and over 50 (HIV^+^: 0.16%, HIV^-^: 0.10%), although the HIV^+^ over 50 years were slightly lower than HIV^+^ under 50 years. In Fig 4C, the HIV-specific CD8^+^IFN-γ T cells median frequencies were higher in HIV^+^ compared with HIV^-^ in under 50 years (HIV^+^: 0.74%, HIV^-^: 0.48%) and a significant increase (p = 0.0171) over 50 years (HIV^+^: 0.63%, HIV^-^: 0.39%), including lower median frequencies in HIV^+^ over 50 age group (0.63%) compared with HIV^+^ under 50 age group (0.74%). These data suggested that the frequencies of HIV-specific CD8^+^IFN-γ T cells decreased with increasing age of HIV^+^ patients. To add further, our HIV^+^ cohort had unchanged CD4 T cell counts and slightly elevated CD8 T cells with increasing age.

**Fig 5:**
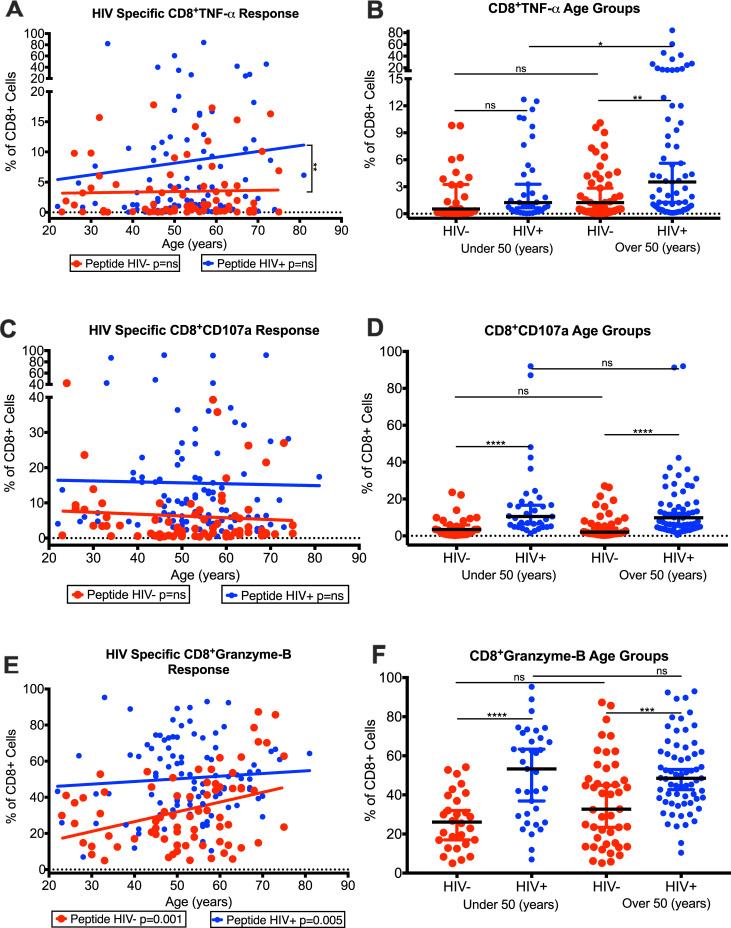
Age related changes in production of TNF-α, Granzyme-B, and CD107a by CD8^+^ T-cells from HIV-infected patients with controlled viremia on ART. Frequencies of HIV Gag/Env peptide pool simulated and unstimulated intracellular TNF-α (A), membrane protein CD107a (C), and intracellular Granzyme-B (E) CD8^+^ T-cell populations with increasing age of HIV^+^ and HIV^-^ cohorts. Frequencies of HIV Gag/Env peptide pool simulated intracellular TNF-α (B), membrane protein CD107a (D), and intracellular Granzyme-B (F) CD8^+^ T-cells in 2 age groups; under 50 years (n = 41 HIV^+^, 35 HIV^-^) and over 50 years (n = 59 HIV^+^, 40 HIV^-^), using interquartile bell curve median HIV^+^ and HIV^-^ cohorts. (n = 100 HIV^+^, n = 75 HIV^-^) (*p<0.05, **p<0.01, ***p<0.001, ****p<0.0001).

Interestingly, there was an increase in the frequencies of HIV-specific CD8^+^TNF-α T-cells in HIV^+^ compared with HIV^-^ with their increasing age, with a significant increase in slope for HIV^+^ than HIV^-^ (Fig 5A). When comparing the interquartile median age groups for CD8^+^TNF-α T-cells, the under 50 years of age group showed an increase in the median frequencies of these cells in HIV^+^ (1.24%) compared with HIV^-^ (0.53%) and a significant (p = 0.0039) increase over 50 years of age group (HIV^+^: 3.53%; HIV^-^: 1.23%) (Fig 5B). In addition, the HIV^+^ over 50 years age group (3.53%) showed a significant (p = 0.0358) increase in CD8^+^TNF-α T-cells compared with HIV^+^ under 50 years group (1.24%) (Fig 5B). These data showed an increased TNF-α production by CD8^+^ T-cells with increasing age of HIV^+^, suggesting continued inflammation.

We also evaluated the production of CD107a and Granzyme-B by CD8^+^ T-cells with increasing age of our HIV^+^. While there was no significant difference in the frequencies of CD8^+^CD107a T cells in response of HIV Gag/Env peptide pools with increasing age of HIV^+^ (Fig 5C), the frequencies of CD8^+^ Granzyme-B produced by CD8 T cells significantly (p = 0.05) increased (Fig 5E) with increasing age of the HIV^+^. When we compared the two populations using the interquartile median age groups, the HIV^+^ for both under 50 and over 50 years age groups showed a significant (p<0.0001) increase in both CD8^+^CD107a (Fig 5D) and CD8^+^Granzyme-B (Fig 5F) T-cells compared with HIV^-^ groups. However, there was a slight decrease in the frequencies of both CD8^+^CD107a (Fig 5D) and CD8^+^Granzyme-B Fig 5F) T-cells in the HIV^+^ over 50 years group (HIV^+^ CD107a: 9.79; Granzyme-B: 48.5) compared with the HIV^+^ under 50 years group (HIV^+^ CD107a: 10.5; Granzyme-B: 53.3). These data suggest that lower levels of HIV-specific CD8 T-cell responses were seen with older age group of HIV^+^.

## Discussion

In this study, we have investigated HIV-specific CD4 and CD8 T-cell responses in HIV-infected patients with controlled viremia (mostly undetectable; <20 to <40 to very low viral load; <60 to <100 HIV RNA copies/mL) and increasing CD4 T-cell counts and age of the HIV^+^ patients. We show that HIV^+^ patients on long-term ART showed immune restoration, including improvement in IFN-γ production by CD4^+^ T-cells in response to HIV-specific and non-specific stimuli and reduction in HIV-specific CD8^+^ T-cell response due to controlled viremia and improved CD4 T-cell counts. Furthermore, we showed that HIV-specific CD8 T-cell responses, including production of IFN-γ and TNF-α and mediators of cytotoxic CD8^+^ T-cells, CD107a and Granzyme-B, decreased with increasing CD4 T-cell counts in our virologically controlled HIV^+^. However, those HIV^+^ patients with controlled viremia but lower CD4 T-cell counts still maintained higher levels of HIV-specific CD8 T-cell responses, which is consistent with earlier studies that found higher CD8^+^ T-cell responses in HIV^+^ with controlled viremia and lack of restored CD4 T-cell counts [8,9]. Our data showed that there was no correlation between undetectable to very low level of viremia and HIV-specific CD4 and CD8 T cell responses. Furthermore, several of these T-cell responses, including production of pro-inflammatory cytokines, IFN-γ and TNF-α, and cytotoxic T-cell mediators, Granzyme-B and CD107a, decreased with increasing age of the HIV^+^ patients. Taken together, these findings demonstrate that HIV-specific CD8 T-cell responses wane in those HIV^+^ who have achieved suppressed viremia and improved functional CD4 T-cell counts, whereas it persists at higher levels in those HIV^+^ who have failed to restore their CD4 T-cell counts. In addition, these HIV-specific CD8 T cell responses decreased in HIV-infected older patients due to reduced immunity [4].

Several studies have reported that HIV^+^ patients with high viral loads, lower CD4 T-cell counts, and high levels of CD8 T-cell activation (IFN-γ, CD38, HLA-DR) showed lower HIV-specific CD4^+^IFN-γ T-cell responses, which decreased with induction of ART [30–32], naïve ART patients [30,33], long-term non-progressors [34,35], and ART treated pediatric patients [36]. Additionally, it has been reported that HIV-specific CD4 T-cell responses (IFN-γ, IL-2, TNF-α) during early infection or untreated infection may predict disease progression and effective viral control [31,32,37,38]. Conversely, our study found a significant increase in HIV-specific IFN-γ production in virologically controlled HIV^+^ on long-term ART with improved CD4 T-cell counts, supporting that these HIV^+^ patients had immune recovery of CD4^+^ T-cells and function [25]. Our data also showed a significant increase overall in HIV-specific CD8^+^IFN-γ T-cells in HIV^+^, however, this response decreased with increasing CD4 T-cell counts. On the hand, our data found no correlation between CD4 and CD8 T cell responses and varying levels of mostly undetectable viral load (<20 to <40 HIV RNA copies/mL) to some with very low viral load (<60 to <100), suggesting that these very low levels of viral RNA copies were not enough to cause further stimulation, as also shown in previous studies [39,40]. Our data suggest that improved CD4 T-cell counts and function play an important role in reducing HIV-specific CD8^+^ T-cells activation that is most likely not needed to contain HIV due to controlled viremia (undetectable to very low viral load) by long term ART.

Our study further examined HIV-specific TNF-α made by CD8^+^ T-cells that has been shown to be maintained at higher levels in ART naïve and non-progressors HIV^+^ patients [33,35], whereas low viral load correlated with reduced HIV-specific CD8^+^ T-cell responses [41]. Our data showed that HIV-specific CD8^+^TNF-α expression decreased with increasing CD4 T-cell counts in HIV^+^, suggesting reduced activation of HIV-specific CD8^+^ T-cells due to controlled viremia. This data was further supported by decreasing levels of CD8^+^ T-cell cytotoxic activation and degranulation markers, Granzyme-B and CD107a, in our HIV^+^ with increasing CD4 T-cell counts. Granzyme-B, a serine protease, performs numerous functions, including mediating apoptosis [28,29,37], inflammation through cytokine release, degrading extracellular matrix proteins and aging [28]. Our data showed a significant increase in overall Granzyme-B expression by CD8^+^ T-cells in our HIV^+^ patients consistent with several recent reports [37,42–44]. However, the significant decline in CD8^+^ Granzyme-B T-cells with increasing CD4 T-cell counts in our HIV^+^, suggest that these cytotoxic functions are not desired with controlled viremia and improved CD4 T-cell counts. Also, CD107a is lysosomal-associated membrane protein-1 and a marker for activation and degranulation of NK cells [45,46], CD4^+^ and CD8^+^ T-cells [45,47] and associated with HIV infection [37,48,49]. Our findings that HIV-specific CD8^+^ T-cells expressing CD107a in HIV^+^ with controlled viremia declined with increasing CD4^+^ T-cell counts, suggests decreased degranulation activation.

Since most of our HIV^+^ patients have aged on long-term ART and achieved controlled viremia (undetectable to very low viral load) and primarily restored CD4^+^ T-cell counts, we also evaluated the effect of aging on T-cell responses as aging is known to modulate the T-cell response [4]. Our data showed that while HIV-specific IFN-γ produced by CD4^+^ T cells remain unchanged with increasing age of HIV^+^, HIV-specific CD8^+^IFN-γ^+^ T-cells decreased with increasing age of HIV^+^ patients, although still higher than HIV^-^ individuals. These data support the notion that aging reduces T cell functions, however, some level of immune activation continues in HIV^+^ patients due to prolonged residual HIV infection and increasing age of the patients [4,50–52], despite controlled viremia. Our data on IFN-γ production by CD4^+^ T-cells in response to HIV-antigens that was unchanged and did not show a decline with increasing age due to reduced immunity in aging [4] probably could be due to the effects of HIV antigen stimulation of a small frequencies of CD4 T cells. However, slightly lower levels of IFN-γ produced by CD4 T cells in response to HIV-antigens were seen in HIV^+^ patients’ above 50 years group than under 50 years group. The age span of our cohorts (both HIV^+^ and HIV^-^) is likely too narrow to adequately reflect the more profound age relevant changes seen in other studies for the CD4 population specifically [53] and if more individuals below 40 and above 65 years of age were present in the cohort, we might have seen a decline in CD4 T cell function with increasing age. Furthermore, TNF-α, a pro-inflammatory cytokine, has been shown to be produced during HIV infection both in ART-naïve and ART-treated patients [54] and aging [55] and maintained at some levels in virologically controlled and restored CD4 T-cell counts of HIV^+^ patients, as also found in our study. We found that HIV-specific TNF-α produced by CD8^+^ T-cells increased with increasing age of HIV^+^ and were significantly higher in over 50 year age group than below 50 years age group, suggesting continued inflammation due to increasing age, prolonged residual HIV-infection and ongoing immune activation [4,50–52]. In addition, the cytotoxic mediators made by CD8 T cells such as CD107a and Granzyme-B were reduced in HIV^+^ over 50 years of age compared with below 50 years of age. It’s important to note that there was no significant difference in the CD4 and CD8 T cell counts with increasing age in our HIV^+^ patients. Our data suggest that some levels of inflammation persists in HIV^+^ with controlled viremia, but increases with increasing age of our HIV^+^, but with increasing age have a reduced level of HIV-specific CD8 T-cell response because of dysregulation of immunity in older individuals [4].

Despite effective control of HIV replication with ART in our HIV^+^ aging patients, immune recovery appears to correlate strongly with increased CD4 T-cell counts. Nevertheless, immune activation and inflammation appear to be maintained in HIV^+^ patients with improved or higher CD4 T-cells counts due to residual HIV persistence, but higher in patients with incomplete CD4 T-cell counts renewal and reduced in older HIV^+^ patients due to immune aging, which may probably improve in the subsequent years of continued ART and better immune reconstitution. This aspect can be assessed in the longitudinal follow up samples of these HIV^+^ collected over time. Defining the pathogenesis of residual viremia, immune dysfunction and incomplete CD4 T-cell recovery may aid in recognizing possible cure or strategies to eliminate HIV from infected patients with controlled or residual viremia.

## Supporting information

S1 Fig Frequencies of HIV Gag/Env peptide pool simulated intracellular IFN-γ (A) in CD4^+^ and (B) in CD8^+^ T-cells with varying levels of undetectable (<20 to <40 HIV RNA copies/mL) to very low viral load (<60 to <100). (C) Frequencies of PMA stimulated intracellular IFN-γ in CD8^+^ T cells in HIV^+^ and HIV^-^ cohorts. (D) Correlation between undetectable to very low viral load and CD4 T cell counts of HIV^+^ patients.
(EPS)
